# Effect of Selected Truffle-Associated Bacteria and Fungi on the Mycorrhization of *Quercus ilex* Seedlings with *Tuber melanosporum*

**DOI:** 10.3390/biotech14030069

**Published:** 2025-09-01

**Authors:** Eva Gómez-Molina, Pedro Marco, Sergi Garcia-Barreda, Vicente González, Sergio Sánchez

**Affiliations:** 1 Centro de Investigación y Experimentación en Truficultura (CIET), Diputación de Huesca, Polígono Fabardo s/n, 22430 Graus, Spain; 2 Departamento de Ciencia Vegetal, Centro de Investigación y Tecnología Agroalimentaria de Aragón (CITA), Avenida de Montañana 930, 50059 Zaragoza, Spain; pmarcomo@cita-aragon.es (P.M.); sgarciaba@cita-aragon.es (S.G.-B.); ssanchezd@cita-aragon.es (S.S.); 3 Instituto Agroalimentario de Aragón-IA2, CITA-Universidad de Zaragoza, 50013 Zaragoza, Spain; 4 Instituto de Ciencias Agrarias, Consejo Superior de Investigaciones Científicas (ICA-CSIC), Calle Serrano 115 b, 28006 Madrid, Spain; vgonzalezg@ica.csic.es

**Keywords:** truffle cultivation, *Tuber melanosporum*, ectomycorrhiza, mycorrhizal seedling, inoculation methods, mycorrhizal helper bacteria

## Abstract

The success of truffle cultivation is especially dependent on the quality of truffle-mycorrhized seedlings, which are typically produced in nurseries under aseptic conditions to avoid root colonization by undesired ectomycorrhizal fungi. However, such practices may also eliminate beneficial microorganisms that could support truffle symbiosis and improve seedling quality. In this study, twelve endophytic bacterial and fungal strains, isolated from the *Tuber melanosporum* environment (gleba tissue, mycorrhizae and truffle *brûlé*), were tested for their effect on *T. melanosporum* mycorrhization levels in inoculated *Quercus ilex* seedlings under nursery conditions. Co-inoculation with a strain of *Agrobacterium tumefaciens* significantly enhanced root colonization by *T. melanosporum*, supporting its potential role as mycorrhizal helper bacterium. In contrast, a strain of *Trichoderma harzianum* negatively affected mycorrhization. The remaining strains did not show significant effects on seedling mycorrhization or seedling growth. Our findings support the hypothesis that specific bacterial strains associated with truffles can act as mycorrhizal helper bacteria, highlighting the potential for co-inoculation strategies to enhance quality of truffle-inoculated seedlings in nurseries. However, further research is needed to gain a deeper understanding of the interactions within the mycorrhizosphere that could contribute to improving nursery seedling quality.

## 1. Introduction

Truffles are the ascocarps of edible fungi belonging to the genus *Tuber*. Among all the species found in Spain, the black truffle (*Tuber melanosporum* Vittad.) is the most economically and gastronomically valuable due to its distinctive aroma. This hypogeous fungus grows wild in symbiosis forming ectomycorrhizae with several forest species of the genus *Quercus*. Nowadays, most of *T. melanosporum* production worldwide is harvested from plantations of inoculated seedlings [1,2], established in areas with suitable edaphoclimatic conditions that allow the fungus to complete its life cycle [3,4]. For these inoculated seedlings, which are produced in specialized nurseries, the mycorrhization level is used as the main indicator of quality, since a higher colonization level of the target species favors its persistence after outplanting, in the face of competition from native soil-borne ectomycorrhizal fungi.

Mycorrhizal symbiosis is currently recognized as more than just a bilateral relationship between fungi and plant roots. Soil-borne bacteria and fungi appear to play a pivotal role in the complex biological processes of nutrient exchange and signaling between soil fungi and plant roots [5,6]. Several bacteria and fungi that have been identified within truffle mycorrhizae, truffle ascocarps or in the soil within their direct influence are thought to harbor plant growth-promoting (PGP) or mycorrhizal helper (MH) activity [7,8,9]. The bacterial community within truffle ascocarps is commonly dominated by *Pseudomonadota*, with species belonging to genera *Bradyrhizobium*, *Pseudomonas*, *Rhizobium*, *Variovorax* and *Ensifer* being frequently detected [5,10,11]. Several taxa from these microbial groups play active roles not only in the mycorrhization process, but also in promoting host plant development. Some Rhizobacteria enhanced nursery plant production, host plant growth and ascocarp yield of *Terfezia* desert truffles [12]. Bradyrhizobia are capable of fixing nitrogen, enhancing plant growth and boosting oxygen production [13,14]. *Pseudomonas* showed broad MH and PGP capabilities [15,16]. Other bacteria which are considered intimately linked to fungal growth have also been identified in ectomycorrhizae or ascocarps from several *Tuber* species, such as those belonging to the genus *Bacillus* in *T. borchii*, *T. aestivum* and *T. melanosporum* [17,18].

These findings have sparked growing interest in the use of specific bacteria to improve the mycorrhizal colonization of truffle-inoculated seedlings in the nursery [15,19]. Several studies have evaluated the effect of selected bacteria on root colonization by *T. melanosporum* in *Corylus avellana* L. [20], *Pinus halepensis* Mill. [19], *Pinus nigra* Arnold [21], *Quercus faginea* Lam. [22] and *Quercus ilex* L. [23] seedlings in the nursery, although they report contrasting effects on truffle mycorrhization levels. These studies evaluated bacterial taxa that are present in truffle soils, mainly belonging to genus *Pseudomonas*, but most of them used commercial strains for their assays. To our knowledge, no study has tested bacterial or fungal strains isolated from the mycorrhizosphere or the ascocarp of *T. melanosporum*, except for Mamoun et al. [20]. In addition, information on *Q. ilex* is scarce, despite it being the main host tree for *T. melanosporum* in Spain and also widely used in France and Italy [24].

The primary objective of this study was to evaluate the ability of 12 endophytic bacteria and fungi isolated from the *T. melanosporum* environment (ten from ascocarp, one from mycorrhiza, and one from a truffle *brûlé*) to enhance the establishment and formation of black truffle mycorrhizae on the roots of *Q. ilex* seedlings in nursery conditions. We also evaluated their effect on seedling growth. We hypothesized that: (i) microbial species living in association with truffles may play a positive role in the establishment of *T. melanosporum* mycorrhizae [5,17] and (ii) the combined inoculation of truffle with these microorganisms could positively influence the growth of *Q. ilex* seedlings. Both the mycorrhizal status of seedlings and the vegetative quality of these seedlings are relevant factors in the overall quality of *T. melanosporum*-inoculated seedlings.

## 2. Materials and Methods

### 2.1. Selection of Microorganisms and Inoculum Preparation

Thirteen microbial strains were selected for the experiment, 12 of which were isolated from black truffle environment and one, *Bradyrhizobium japonicum* (BJ, DSM accession number: 30131), was purchased from the Spanish Type Culture Collection (University of Valencia, Spain). Of these, 11 were bacterial strains and two were fungal taxa (Table 1). We included the BJ strain in our experiment because *Bradyrhizobium* is the most frequently detected genus in ascocarps of hypogeous fungi [10]. The ten strains isolated from truffle gleba were obtained from unripe (July to December) and ripe (December to March) ascocarps sampled in a truffle plantation in eastern Spain [25]. For the isolation, gleba samples from the different ascocarps were carefully collected to avoid contamination from the peridium, homogenized in sterile water (1.5 mL tubes) using sterile micropestles and plated on PCA medium (Plate Count Agar, Avantor Sciences, Radnor, PA, USA). Purity of the isolates was checked by streaking on agar plates. Pure cultures obtained were identified through sequence-based methods. DNA from each strain was extracted using the REDExtract-N-Amp^TM^ Plant PCR Kit (Sigma-Aldrich, St. Louis, MO, USA) as follows. An aliquot of fresh (1-day-old) pure culture was suspended in 50 μL of Extraction Solution^TM^ (Sigma-Aldrich, USA), vortexed and incubated at 95 °C for 10 min. Then, 50 μL of Dilution Solution^TM^ (Sigma-Aldrich, USA) was added and the tubes were centrifuged at 13,000 rpm for 5 min. A volume of 2.5 μL of the supernatant was added to a PCR mix containing: 14 μL of PCR grade water, 5 μL of 1X MyTaq^TM^ Reaction Buffer (Bioline, London, UK), 1 μL of 1% (*w*/*v*) Bovine Serum Albumin (Sigma-Aldrich, USA), 1 μL of 10 μM universal primers 8F and 1492R (synthetized by Stab Vida, Caparica, Portugal) and 0.5 μL of 5 U μL^−1^ MyTaq^TM^ DNA Polymerase (Bioline, London, UK). Thermocycling profile was 94 °C for 2 min; 33 cycles of 94 °C for 30 s, 51 °C for 1 min and 72 °C for 1 min, followed by a final extension at 72 °C for 7 min. Each PCR reaction included its own positive and negative controls. Amplicons were visualized on a 1.7% (*w*/*v*) agarose gel stained with SYBR Safe^TM^ DNA Gel Stain (Thermo Fisher Scientific, Waltham, MA, USA), purified using the QIAquick^®^ PCR Purification Kit (Qiagen, Hilden, Germany) and sent for sequencing (Stab vida, Caparica, Portugal). Quality of the obtained sequences was assessed, and low-quality edges were removed using 4Peaks software (Version 1.8; Griekspoor A. and Groothuis T., https://nucleobytes.com/4peaks, accessed on 10 March 2025) [26]. All sequences were registered in the NCBI GenBank^®^ database (http://www.ncbi.nlm.nih.gov/nucleotide, accessed on 17 March 2025) [27]. Bacterial identification was carried out by searching highly similar sequences in the GenBank database using the megablast procedure and default settings.

For the inoculum preparation, all microorganisms were maintained as pure cultures incubated at 26 °C in darkness. Bacterial strains were grown in TSB-YE (Triptic Soy Broth, Oxoid, Basingstoke, UK, with 0.6% Yeast Extract) at 200 rpm for 3 days, reaching 1–3 × 10^9^ CFU mL^−1^, as confirmed by plate counts on TSA-YE (Triptic Soy Broth, Oxoid, Basingstoke, UK, with 0.6% Yeast Extract and 2% agar). The *Tulasnella tubericola* strain (TT) was cultivated in PDB (Potato Dextrose Broth, Oxoid, Basingstoke, UK) at 200 rpm for 4 days, yielding ≈ 10^7^ somatic mycelial propagules mL^−1^ (mostly molinioid cells), quantified on DRBC agar (Dichloran Rose Bengal Chloramphenicol agar, Avantor Sciences, Radnor, PA, USA). The *Trichoderma harzianum* strain (TH) was grown in PDA (Potato Dextrose Agar, Oxoid, UK) for four days until conidia fully covered the plates. Conidia were collected using sterile distilled water and the suspension was adjusted to 3 × 10^8^ conidia mL^−1^, as determined with a Neubauer haemocytometer. The day before inoculation, all strains were encapsulated in alginate beads, prepared according to Buzón-Durán et al. [28]. Briefly, 13 mL of each microorganism suspension was added to 120 mL of sterile 2% sodium alginate solution (*w*/*v*). This solution was gradually dispensed drop by drop into a 3% calcium carbonate solution to form beads, in constant agitation for 45 min until the hydrogels were completely cured. Inoculations were performed 24 h later, with the beads kept at 6 °C until their use.

**Table 1:** Microbial strains selected for the study: species name, abbreviations used along this work, strain origin, isolation and culturing media for inoculations, PCR primers used for sequence-based identifications and Genbank accession numbers.

Species	Abbreviation	Origin	Isolation/Culturing Media	Primers/Genbank Accession Number
*Tulasnella tubericola*	TT	Isolated from *T. melanosporum* mycorrhiza [29]	PDA/PDB	ITS1-ITS4/KX929166
*Trichoderma harzianum* (T50)	TH	Isolated from soil inside a *T. melanosporum brûlé* [30]	TSM (Trichoderma Selective Medium)/PDA	ITS1F-ITS4/KX343087
*Bradyrhizobium japonicum* (DSM 30131)	BJ	Purchased from the Spanish Type Culture Collection, isolated from *Glycine hispida* nodules in Japan	-/TSB-YE	-/NCBI reference sequence: NR_119191
*Variovorax* sp.	Vsp	Isolated from unripe *T. melanosporum* gleba (August 2017)	PCA/TSB-YE	8F-1492R/PV297981
*Variovorax paradoxus*	VP	Isolated from ripe *T. melanosporum* gleba (December 2017)	PCA/TSB-YE	8F-1492R/PV297985
*Ensifer adhaerens* (strain 1)	EA1	Isolated from unripe *T. melanosporum* gleba (August 2017)	PCA/TSB-YE	8F-1492R/PV297983
*Ensifer adhaerens* (strain 2)	EA2	Isolated from ripe *T. melanosporum* gleba (February 2018)	PCA/TSB-YE	8F-1492R/PV297989
*Agrobacterium tumefaciens* (strain 1)	AT1	Isolated from unripe *T. melanosporum* gleba (August 2017)	PCA/TSB-YE	8F-1492R/PV297982
*Agrobacterium tumefaciens* (strain 2)	AT2	Isolated from ripe *T. melanosporum* gleba (January 2018)	PCA/TSB-YE	8F-1492R/PV297986
*Kocuria rhizophila* (strain 1)	KR1	Isolated from unripe *T. melanosporum* gleba (July 2017)	PCA/TSB-YE	8F-1492R/PV297980
*Kocuria rhizophila* (strain 2)	KR2	Isolated from ripe *T. melanosporum* gleba (January 2018)	PCA/TSB-YE	8F-1492R/PV297988
*Pseudomonas* sp. (strain 1)	Psp1	Isolated from unripe *T. melanosporum* gleba (September 2017)	PCA/TSB-YE	8F-1492R/PV297984
*Pseudomonas* sp. (strain 2)	Psp2	Isolated from ripe *T. melanosporum* gleba (January 2018)	PCA/TSB-YE	8F-1492R/PV297987

### 2.2. Experimental Design

Mature *T. melanosporum* ascomata used as inoculum were harvested from different truffle orchards in Huesca province (northern Spain) and taxonomically identified based on morphological features [31]. The ascomata were surface-cleaned with a brush under cool water, then surface-sterilized by immersion in 70% ethanol and flamed. After sterilization, they were thinly sliced and air-dried at room temperature for 7 days until fully dehydrated. Complete desiccation was confirmed by the brittle texture of the tissue, which fractured easily under slight mechanical pressure. The dried material was then homogenized into a fine powder using a coffee grinder.

The *Q. ilex* acorns were sourced from the Spanish provenance region of Sistema Ibérico (acquired from Centro Nacional de Recursos Genéticos Forestales). In January 2018, they were surface-sterilized by immersion in a 5% sodium hypochlorite solution for 60 min and then germinated in a vermiculite tray, which was also previously disinfected. By June 2018, when most seedlings had developed 6–8 leaves and lateral roots, they were carefully removed from the tray, mechanically root-pruned at the tap root end to eliminate any root-architecture defects. Seedlings with malformations, poor development or scarce fine roots were excluded. Immediately after removing the seedlings from the tray, they were transplanted into pots (Full-pot^®^, Acudam, Lleida, Spain, 450 mL, 18.5 cm deep, 25 cm^2^ top area) and simultaneously inoculated with the selected microbial strains and *T. melanosporum*. Microbial inoculation was carried out by mixing 2.5 mL of alginate beads per pot into the potting substrate (Profi-Substrat^®^, Gramoflor, Germany, 60% Sphagnum white peat, 40% Sphagnum black peat, with pH adjusted to 7.5 with dolomite). In order to minimize contamination, seedlings were cultivated under standard management practices established for truffle nurseries and were maintained physically distant from contamination sources [32]. Two control treatments were included: a procedural control (P-Con) to test the effect of the target microorganims and an absolute control (A-Con) to assess the influence of alginate beads addition with respect to the P-Con. In the P-Con, alginate beads without microbial content were added to the substrate, while in the A-Con, no beads were added. Truffle inoculation was performed by root-powdering with a talcum (hydrated magnesium silicate) powder carrier, following Garcia-Barreda et al. [33] with the inoculum adjusted to a rate of 1.5 g of fresh truffle per seedling. Twelve replicates per treatment were prepared. Additionally, five seedlings per microorganism and control were grown without truffle inoculum, to ensure that truffle mycorrhizae resulted solely from the artificial inoculation.

The plants were kept in the CIET greenhouse in Graus (Huesca province, NE Spain) and sprinkle irrigated to saturation 2–3 times per week during summer and once a week during winter. Maximum temperatures occurred in August 2018 (daily mean 25.7 °C, absolute maximum 40.2 °C), while minimum temperatures were recorded in February 2018 (daily mean 6.8 °C, absolute minimum −6.6 °C).

### 2.3. Seedling Measurement and Assessment of T. melanosporum Colonization

In April 2019, the stem length and root collar diameter of seedlings were measured, as widely used attributes in forest seedling quality assessment [34]. After carefully removing the substrate from the root systems by washing, the entire root balls of the seedlings were stored at −20 °C until further analysis. The root colonization by ectomycorrhizal fungi was assessed using the INIA-Aragón method, a morphological analysis that enables evaluation of the variability along the depth profile [35]. The root system of each seedling was divided into three segments of roughly the same length (corresponding to 0–6 cm, 6–12 cm and 12–18.5 cm depth) and root fragments were randomly collected from each segment. For each depth segment, at least 100 root tips were counted and classified as non-mycorrhized or mycorrhized, with the latter further classified into *T. melanosporum* or contaminant morphotypes following Rauscher et al. [36] and Agerer [37]. The only contaminant morphotype that was found was *Sphaerosporella brunnea* (Alb. & Schwein.) Svrček & Kubička. Four seedlings, one from the *Variovorax paradoxus* (VP) treatment, two from the *Agrobacterium tumefaciens* strain 1 (AT1) treatment and one from the *Kocuria rhizophila* strain 2 (KR2) treatment, were excluded due to poor seedling growth and lack of root tips, resulting in a final sample size of 176.

### 2.4. Data Analysis

The effect of the inoculated microorganisms on the percent root colonization by *T. melanosporum* was analyzed using a generalized linear model (GLM). Due to overdispersion from the binomial error distribution, a quasibinomial distribution was applied. The proportion of seedlings in which the contaminant *S. brunnea* was present was analyzed using a generalized (binomial) linear model. The stem length and root collar diameter of the truffle-inoculated seedlings were analyzed with linear models, with stem length log-transformed to meet model assumptions (homogeneity of variance, normality and linearity). Significant differences among treatments were identified using a least squares means test, with a significance threshold of *p* = 0.05. The distribution of *T. melanosporum* colonization level along the depth profile was analyzed with a linear mixed model, considering each depth segment as a different sample and treating depth as a repeated measures variable. All analyses were performed in R version 4.4.1 (RCore Team, Vienna, Austria) using the emmeans package version 1.9.7and nlme package version 3 [38,39,40].

## 3. Results

**Figure 1:**
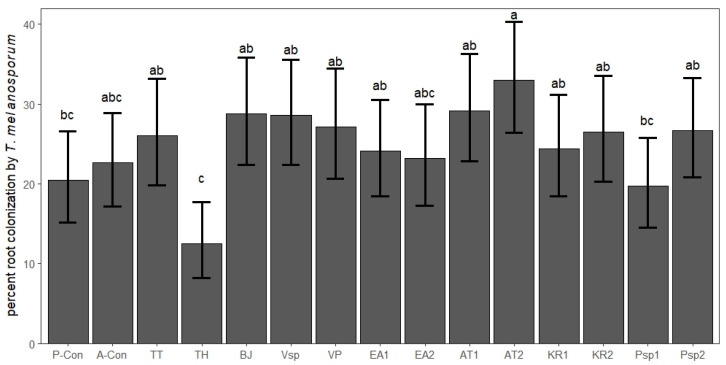
Percent root colonization by *Tuber melanosporum* in the seedlings co-inoculated with *T. melanosporum* and microorganisms from truffle ascocarps and mycorrhizae (predicted values and 95% confidence intervals, different letters indicate significant differences according to the least square means test, α = 0.05, *n* = 176). P-Con: procedural control, A-Con: absolute control, TT: *Tulasnella tubericola*, TH: *Trichoderma harzianum*, BJ: *Bradyrhizobium japonicum*, Vsp: *Variovorax* sp., VP: *Variovorax paradoxus*, EA1: *Ensifer adhaerens* (strain 1), EA2: *Ensifer adhaerens* (strain 2), AT1: *Agrobacterium tumefaciens* (strain 1), AT2: *Agrobacterium tumefaciens* (strain 2), KR1: *Kocuria rhizophila* (strain 1), KR2: *Kocuria rhizophila* (strain 2), Psp1: *Pseudomonas* sp. (strain 1), Psp2: *Pseudomonas* sp. (strain 2).

Ten months after inoculation, *T. melanosporum* showed an average root colonization of 22.8% (standard deviation, SD: 11.5). All the seedlings presented *T. melanosporum* mycorrhizae except for two seedlings in the TH treatment, one in the BJ treatment and three in the *Ensifer adhaerens* strain 2 (EA2) treatment. The GLM revealed that at least one of the co-inoculated microorganisms had a significant effect on the root colonization by *T. melanosporum* in *Q. ilex* seedlings (F = 6553, *p*-value = 0.003, *n* = 176). The post hoc analysis indicated that the AT2 treatment was the only one with significantly higher truffle mycorrhizal levels than the P-Con, while the TH treatment exhibited significantly lower values compared to most of the other co-inoculation treatments (Figure 1, Table S1).

The only contaminant ectomycorrhizae present in the samples were those of *S. brunnea* (*Pyronemataceae*, *Pezizales*) [41]. This fungus appeared in 3.4% of the truffle-inoculated seedlings, with an average root colonization value of 0.2% (SD: 1.6). The GLM analysis showed that the frequency of *S. brunnea* occurrence in the *T. melanosporum*-inoculated seedlings was not significantly affected by any of the co-inoculated microorganisms (z-value = 0.002, *p* = 0.99, *n* = 176, Table S2). The additional non-truffle-inoculated seedlings (*n* = 60) also presented *S. brunnea* mycorrhizae (Figure S1), but they did not show mycorrhizae of *T. melanosporum* or any other ectomycorrhizal fungi.

**Figure 2:**
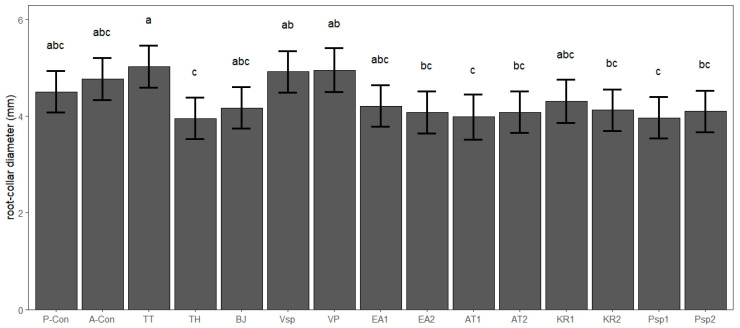
Root-collar diameter of the *Quercus ilex* seedlings co-inoculated with *Tuber melanosporum* and a series of microorganisms from truffle ascocarps and mycorrhizae (predicted values and 95% confidence intervals, different letters indicate significant differences according to the least square means test, α = 0.05, *n* = 176). P-Con: procedural control, A-Con: absolute control, TT: *Tulasnella tubericola*, TH: *Trichoderma harzianum*, BJ: *Bradyrhizobium japonicum*, Vsp: *Variovorax* sp., VP: *Variovorax paradoxus*, EA1: *Ensifer adhaerens* (strain 1), EA2: *Ensifer adhaerens* (strain 2), AT1: *Agrobacterium tumefaciens* (strain 1), AT2: *Agrobacterium tumefaciens* (strain 2), KR1: *Kocuria rhizophila* (strain 1), KR2: *Kocuria rhizophila* (strain 2), Psp1: *Pseudomonas* sp. (strain 1), Psp2: *Pseudomonas* sp. (strain 2).

The seedlings inoculated with *T. melanosporum* presented an average stem length of 11.7 cm (SD: 3.1) and an average root-collar diameter of 4.3 mm (SD: 0.8). According to the linear model, the stem length was not significantly affected by any of the co-inoculated microorganisms (F = 1.72, *p* = 0.056, *n* = 176). The root-collar diameter showed significant differences among some of the co-inoculated microorganisms (F = 3.07, *p* < 0.001, *n* = 176), although none of the co-inoculation treatments showed significant differences with any of the control treatments (Figure 2).

The effect of microorganism co-inoculation on the depth distribution of *T. melanosporum* mycorrhizae was analyzed using a linear mixed model, but no significant interaction between the co-inoculated microorganisms and the depth segments was found (F = 1.16, *p* = 0.27, *n* = 528). The percent root colonization by *T. melanosporum* was significantly affected by the depth segment (F = 214.84, *p* < 0.001, *n* = 528), with all co-inoculation treatments showing decreasing colonization with depth (Figure S2).

## 4. Discussion

Our results indicate that co-inoculation of *T. melanosporum* with the strain AT2 resulted in significantly higher truffle colonization levels compared to the P-Con (addition of alginate beads without microbial content). A second *A. tumefaciens* strain (AT1) was also tested and showed a marginally significant positive effect on truffle mycorrhization (*p* = 0.055, Table S1). *Agrobacterium tumefaciens* is a ubiquitous soil-borne bacterium typically associated with roots, tubers or underground stems [42]. The ability of other *A. tumefaciens* strains to transfer T-DNA and induce plant growth regulators [43] has significant applications in agriculture, supporting its potential for enhancing mycorrhizal plant performance [44,45].

Recent studies have reclassified all species previously grouped under the genus *Agrobacterium*, assigning most of them to *Rhizobium* and splitting others to *Ruegeria*, *Pseudorhodobacter* and *Stappia* [46]. The genus *Rhizobium* and other *Rhizobiaceae* members are well recognized in the plant microbiome for their PGP properties [47,48] and roles as MH bacteria [49]. Our BLAST analysis (version 2.16.0) failed to accurately classify strain AT2, as it showed the maximum score of similarity with several strains from *Agrobacterium* and *Rhizobium* genera. Although the phylogenetic reconstruction supported its classification as *A. tumefaciens,* a whole-genome sequencing will help to confirm its identity in the future. Other recent works based on the comparison of molecular data (multi-locus phylogenies, genomic annotation, etc.) have revealed the heterogeneity and complexity of this genus, with the existence of genomospecies [50] that are especially abundant within the *A. tumefaciens* complex. This view suggests that the collective species includes both pathogenic and non-pathogenic (*Rhizobium*-like) microorganisms (e.g., *Agrobacterium radiobacter*), as well as species for which the type of relationship established with their plant hosts is not fully understood, as is the case of *Agrobacterium* (*Rhizobium*) *pusense*. Our strain AT2 exhibited MH capabilities when co-inoculated with *T. melanosporum*, which are also found in *Rhizobium* [47] and specifically in *Rhizobium pusense* [51]. This supports its potential as a promising candidate to improve the quality of black truffle-mycorrhized seedlings under nursery conditions. However, the precise mechanisms by which strain AT2 contributes to the process remain to be elucidated, as MH bacteria can act in multiple ways: mobilizing soil nutrients, fixing atmospheric nitrogen, producing growth factors and protecting the root–fungus system against pathogens [49]. These diverse activities may induce spore germination, enhance hyphal growth, promote root branching and root–fungus contacts, and mitigate chemical or biological stresses [49], thus supporting the overall positive effect on mycorrhizal establishment.

Our study did not show any significant effect for two native *Pseudomonas* strains on *T. melanosporum* mycorrhization levels. The first studies about *Pseudomonas* effect on truffle mycorrhization showed a negative temporary effect of strains isolated from truffle soils [20]. Several studies with the *P. fluorescens* strain CECT 844 showed contrasting effectiveness as MH bacterium: a positive effect with *P. halepensis* and nonsignificant effects with *P. nigra* and *Q. faginea* [19,21,22]. Giorgi et al. [23] found a positive effect of a *Pseudomonas* strain on *Q. ilex* colonization by *T. melanosporum*, whereas Piñuela et al. [22] did not find any significant effect with *Pseudomonas putida* on *Q. faginea*. Our nonsignificant results for native strains of *Pseudomonas* and the contrasting results of other studies for this genus suggest a limited interest in improving the quality of truffle seedlings in the nursery. However, previous studies also showed that inoculation with the *P. fluorescens* strain Aur6 improved both growth and drought tolerance in *P. halepensis* and *Quercus coccifera* L [52,53]. The improvement of host plant vigor and resistance to stress, which are often more clearly observed under adverse conditions such as nutrient-limitation [54], would also be interesting for the development of truffle plantations in the field. In this regard, it would also be interesting to assess the PGP capabilities of the truffle-associated microorganisms.

On the other hand, our study showed marginally significant positive effects of the *Variovorax* (Vsp) and the commercial BJ strains on *T. melanosporum* mycorrhization (*p* = 0.069 and *p* = 0.068, respectively, Table S1). It would be worth evaluating whether other native strains of *Bradyrhizobium* or *Variovorax* could show MH abilities. These genera are amongst the most frequently detected within truffle ascocarps and mycorrhizae [5,10]. Despite this, Giorgi et al. [23] found that co-inoculating a *Bradyrhizobium* strain with *T. melanosporum* did not significantly improve truffle mycorrhization and decreased fine root density.

The tested TH strain presented significantly lower *T. melanosporum* colonization than most of the remaining microbial treatments, contrary to a previous study where it significantly improved truffle colonization [55]. The inhibitory effect we found is in line with the fact that *T. harzianum* is widely recognized for its fungicidal activity and its role as a biocontrol agent, primarily due to its extracellular chitinase activity, the production of antibiotics such as gliotoxin and other secondary metabolites [56], as well as for its hyperparasitic behavior and its ability for early and massive colonization of the rhizosphere and other plant organs and tissues [57]. Werner et al. [58] questioned the use of *Trichoderma* spp. as bio-control agents in forest nurseries, as a direct result of their antagonism towards ectomycorrhizal colonization. These results are also consistent with previous experiments in which several *T. harzianum* strains native to truffle soils (including the TH strain) showed in vitro inhibitory effects on the mycelial growth of *Armillaria mellea* [59] and *S. brunnea*, although for the latter in vivo effects were not significant [30,60].

Our results did not show that *T. melanosporum*-associated bacteria and fungi generally exhibit MH abilities. Previous studies have reported the effectiveness of bacterial co-inoculation with certain mycorrhizal fungi [19,61,62], showing that adhesion and colonization by MH bacteria on the mycorrhizal surface can enhance both the symbiotic relationship and the pre-symbiotic stages, benefiting the host plant [49]. Our nonsignificant results for many taxa could be related to the fact that they do not play significant roles in mycorrhiza formation or plant growth promotion. Alternatively, these taxa could be involved in specific metabolic or physiological processes that are not directly reflected in mycorrhizal rates or seedling size. It is also possible that the participation of several taxa is needed for an effective impact on truffle mycorrhization, as suggested by Giorgi et al. [23].

We acknowledge that the influence of each microorganism could be more accurately evaluated under gnotobiotic conditions, because of the uncontrolled presence of microbial populations in the peat-based substrate and in the greenhouse environment. *Sphaerosporella brunnea* has been previously reported in peat moss [63]. However, our aim was to reflect standard nursery practices where, in terms of ectomycorrhizal colonization, truffle seedling quality depends on the proportion of fine roots colonized by fungi that successfully form ectomycorrhizae. The A-Con, P-Con and microbial treatments were applied to seedlings from the same germination tray, using identical substrate, inoculated on the same day and cultivated under homogeneous conditions. Moreover, no significant differences in *S. brunnea* colonization rates were found among treatments. Therefore, it is reasonable to attribute the observed differences in *T. melanosporum* colonization to the microbial treatments themselves.

To conclude, we identified an *A. tumefaciens* strain isolated from the gleba of *T. melanosporum* that enhanced the mycorrhization of *Q. ilex* roots by *T. melanosporum* under nursery conditions. This supports the hypothesis that some microorganisms naturally present in the mycorrhizosphere of truffles may act as MH bacteria. However, *T. melanosporum* colonization was significantly different among the evaluated microbial taxa. The TH strain showed lower values than many of the other microorganisms, whereas some rhizobacterial strains (from genera *Pseudomonas*, *Ensifer* or *Variovorax*) did not show any significant effect on either truffle mycorrhization or seedling growth. In the framework of the current truffle nursery practices, under highly controlled and aseptic conditions that reduce the diversity of native mycorrhizosphere microorganisms, our results highlight that the controlled addition of specific microbial strains with MH ability could enhance inoculated seedling quality and symbiotic efficiency under routine nursery production conditions.

## Data Availability

The original data presented in the study are openly available in FigShare at https://doi.org/10.6084/m9.figshare.29327627.v1.

## Supplementary Materials

The following supporting information can be downloaded at: https://www.mdpi.com/article/10.3390/biotech14030069/s1, Figure S1: Frequency of occurrence of *S. brunnea* in seedlings inoculated and not inoculated with *T. melanosporum*. Figure S2: Mycorrhization levels of *T. melanosporum* along pot depth. Table S1: ANOVA table for the analysis of *T. melanosporum* mycorrhization levels. Table S2: ANOVA table for the analysis of *S. brunnea* occurrence frequency.
