# Supplementary material for: Effect of Selected Truffle-Associated Bacteria and Fungi on the Mycorrhization of Quercus ilex Seedlings with Tuber melanosporum
Source: BioTech (Basel). 2025 Sep 1;14(3):69. doi: 10.3390/biotech14030069 (PMC12452314; doi:10.3390/biotech14030069)
Supplement: Supplementary file 1 [file biotech-14-00069-s001.zip › biotech-3787797-supplementary.pdf]

BioTech

Supplementary Material: Effect of Selected Truffle-Associated Bacteria and Fungi on the Mycorrhization of *Quercus ilex* Seedlings with *Tuber melanosporum*

Eva Gómez-Molina, Pedro Marco, Sergi Garcia-Barreda, Vicente González and Sergio Sánchez

Table S1. ANOVA table for the analysis of *T. melanosporum* mycorrhization levels

Table S2. ANOVA table for the analysis of *S. brunnea* occurrence frequency

Figure S1. Frequency of occurrence of *S. brunnea* in seedlings inoculated and not inoculated with *T. melanosporum*

Figure S2. Mycorrhization levels of *T. melanosporum* along pot depth

Table S1. ANOVA table for the analysis of the percent root mycorrhization by *Tuber melanosporum* in the seedings co-inoculated with a series of selected microorganisms (α = 0.05, n=176).

	Estimate	Std. Error	t value	Pr(>|t|)
Intercept [1]	0.452	0.032	14.0	<0.001
Absolute control	0.023	0.045	0.5	0.61
Tulasnella tubericola	0.058	0.046	1.3	0.21
Trichoderma harzianum	-0.098	0.047	-2.1	**0.039**
Bradyrhizobium japonicum	0.084	0.045	1.8	0.068
Variovorax sp.	0.083	0.045	1.8	0.069
Variovorax paradoxus	0.069	0.046	1.5	0.15
Ensifer adhaerens (strain 1)	0.039	0.045	0.9	0.39
Ensifer adhaerens (strain 2)	0.029	0.046	0.6	0.54
Agrobacterium tumefaciens (strain 1)	0.088	0.045	1.9	0.055
Agrobacterium tumefaciens (strain 2)	0.122	0.045	2.7	**0.007**
Kocuria rhizophila (strain 1)	0.041	0.046	0.9	0.37
Kocuria rhizophila (strain 2)	0.063	0.046	1.4	0.18
Pseudomonas sp. (strain 1)	-0.008	0.046	-0.2	0.86
Pseudomonas sp. (strain 2)	0.064	0.045	1.4	0.15

[1] The procedural control was used as statistical control (intercept) for the GLM.

Table S2. ANOVA table for the analysis of the relative frequency of occurrence of *Sphaerosporella brunnea* ectomycorrhizae in the seedings co-inoculated with *T. melanosporum* and microorganisms (α = 0.05, n=176).

	Estimate	Std. Error	z value	Pr(>|z|)
Intercept [1]	-21.6	8440	-0.003	1
Absolute control	19.2	8440	0.002	1
Tulasnella tubericola	20.5	8440	0.002	1
Trichoderma harzianum	$< 10^{-6}$	11900	<0.001	1
Bradyrhizobium japonicum	$< 10^{-6}$	11900	<0.001	1
Variovorax sp.	$< 10^{-6}$	11900	<0.001	1
Variovorax paradoxus	$< 10^{-6}$	12200	<0.001	1
Ensifer adhaerens (strain 1)	$< 10^{-6}$	11900	<0.001	1
Ensifer adhaerens (strain 2)	19.2	8440	0.002	1
Agrobacterium tumefaciens (strain 1)	$< 10^{-6}$	12500	<0.001	1
Agrobacterium tumefaciens (strain 2)	$< 10^{-6}$	11900	<0.001	1
Kocuria rhizophila (strain 1)	$< 10^{-6}$	12200	<0.001	1
Kocuria rhizophila (strain 2)	$< 10^{-6}$	11900	<0.001	1
Pseudomonas sp. (strain 1)	$< 10^{-6}$	11900	<0.001	1
Pseudomonas sp. (strain 2)	19.2	8440	0.002	1

[1] The procedural control was used as statistical control (intercept) for the GLM.

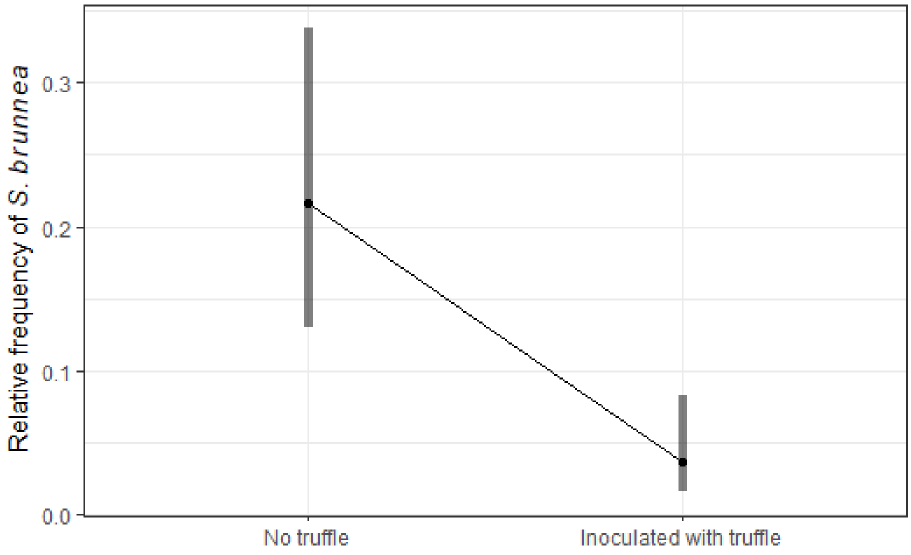

Figure S1. Relative frequency of occurrence of *Sphaerosporella brunnea* ectomycorrhizae in the seedings with and without *T. melanosporum* inoculation (predicted values and 95% confidence intervals), according to the general linear model (F = 16.4, P < 0.001, n = 200). Only the co-inoculation treatments in which both truffle-inoculated and not inoculated seedlings were available are included (seedlings without truffle from the absolute control, the procedural control and the *Variovorax sp.* treatment were lost and thus these co-inoculation treatments were not included in the analysis).

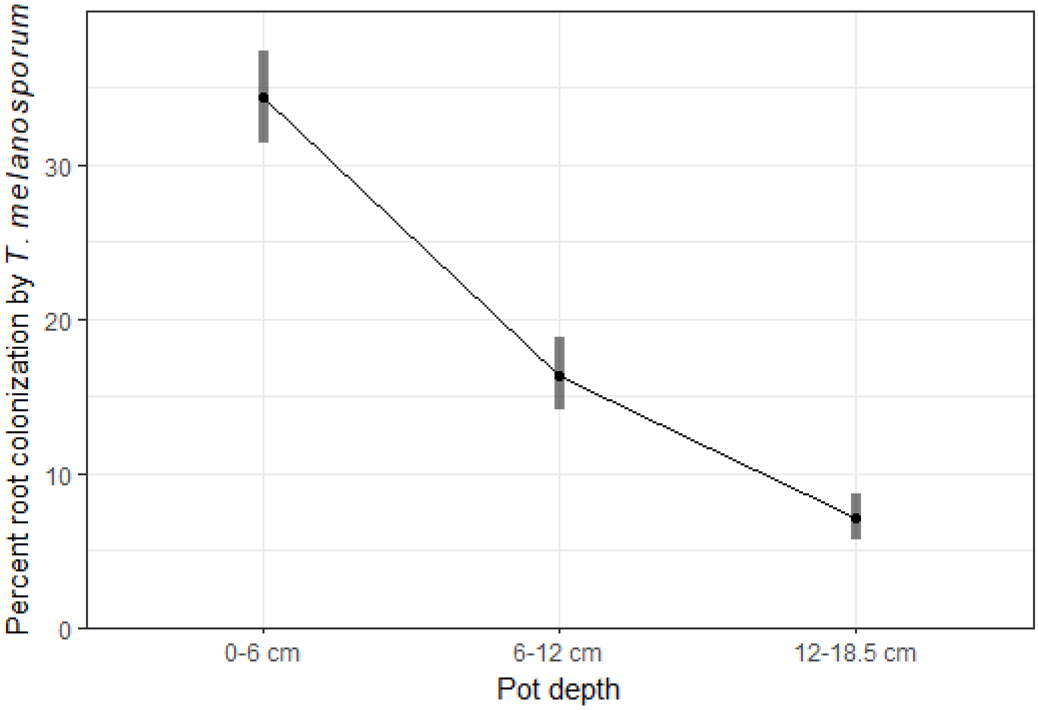

Figure S2. Percent root colonization along the pot depth, according to the linear mixed model (predicted values and 95% confidence intervals, n = 528).
